# Performance of non-invasive respiratory function indices in predicting clinical outcomes in patients hospitalized for COVID-19 pneumonia in medical and sub-intensive wards: a retrospective cohort study

**DOI:** 10.1007/s11739-021-02922-6

**Published:** 2022-01-28

**Authors:** Filippo Cattazzo, Francesco Inglese, Andrea Dalbeni, Salvatore Piano, Martino Francesco Pengo, Martina Montagnana, Davide Dell’Atti, Francesco Soliani, Andrea Cascella, Stefano Vicini, Carmine Gambino, Pietro Minuz, Roberto Vettor, Gianfranco Parati, Paolo Angeli, Cristiano Fava, COVID Internal Medicine Team

**Affiliations:** 1 Internal Medicine and Covid Unit, Department of Medicine, University of Verona, Verona, Italy; 2 Intensive Care Respiratory Unit, Mantua Hospital, Mantua, Italy; 3 Department of Medicine, University and Hospital of Padua, Padua, Italy; 4 IRCCS Istituto Auxologico Italiano, Department of Cardiovascular, Neural and Metabolic Sciences, San Luca Hospital, Milan, Italy; 5 Department of Neurosciences, Biomedicine and Movement Sciences, Clinical Biochemistry Section, University of Verona, Verona, Italy

**Keywords:** COVID-19 pneumonia, Non-invasive respiratory function indices, Clinical outcomes

## Abstract

Coronavirus disease 2019 (COVID-19) is a newly recognized infectious disease which can lead to acute respiratory distress syndrome requiring ventilatory support and intensive care unit admission. The aim of our study is to evaluate the performance of two non-invasive respiratory function indices (the ROX index and the SatO2/FiO2 ratio), as compared to the traditional PaO2/FiO2 ratio, in predicting a clinically relevant composite outcome (death or intubation) in hospitalized patients for COVID-19 pneumonia. Four hospital centers in Northern Italy conducted an observational retrospective cohort study during the first wave of COVID-19 pandemic. Four hundred and fifty-six patients with COVID-19 pneumonia admitted to medical or sub-intensive wards were enrolled. Clinical, laboratory, and respiratory parameters, for the calculation of different indices, were measured at hospital admission. In medical wards (Verona and Padua) the PaO2/FiO2 ratio, ROX index and SatO2/FiO2 ratio were able to predict intubation or death with good accuracy (AUROC for the PaO2/FiO2 ratio, ROX index and SatO2/FiO2 ratio of 75%, 75% and 74%, respectively). Regarding sub-intensive wards (Milan and Mantua), none of the three respiratory function indices was significantly associated with the composite outcome. In patients admitted to medical wards for COVID-19 pneumonia, the ROX index and the SatO2/FiO2 ratio demonstrated not only good performance in predicting intubation or death, but their accuracy was comparable to that of the PaO2/FiO2 ratio. In this setting, where repeated arterial blood gas tests are not always feasible, they could be considered a reliable alternative to the invasive PaO2/FiO2 ratio.

**Supplementary Information:**

The online version contains supplementary material available at 10.1007/s11739-021-02922-6.

## Introduction

Coronavirus disease 2019 (COVID-19) is a newly recognized infectious disease caused by severe acute respiratory syndrome coronavirus 2 (SARS-CoV-2) which has rapidly spread around the world reaching pandemic proportions [1]. Up to 20% of patients develop acute respiratory distress syndrome (ARDS), which may require different grades of oxygen therapy up to ventilatory support and intensive care unit (ICU) admission [2–4]. During the COVID-19 outbreak in Italy, patients not immediately requiring ICU were hospitalized either in medical wards, where oxygen could be primarily delivered with Venturi masks and masks with reservoir bags, or in sub-intensive wards, where high-flux nasal oxygen (HFNC), as well as continuous positive airway pressure (cPAP) and non-invasive ventilation (NIV) were available. These patients often needed to be transferred urgently to the ICU for mechanical ventilation because of respiratory failure refractory to oxygen therapy or NIV [5–8].

Arterial blood gas (ABG) test is considered the gold standard for the diagnosis of respiratory failure; however, especially in medical wards, such test is not always feasible and available. The ABG test allows the assessment of the ratio between arterial partial pressure of oxygen (PaO2, expressed in mmHg) and fractional inspired oxygen concentration (FiO2) (PaO2/FiO2 ratio), which is a validated index of hypoxaemia and is associated with adverse outcomes in patients with respiratory failure [9, 10]. The PaO2/FiO2 ratio is widely used in clinical practice especially for the diagnosis of ARDS according to the Berlin definition [11]. Furthermore, the PaO2/FiO2 ratio helps in patients risk stratification identifying three different categories of ARDS severity (mild, moderate, and severe) [11].

The peripheral blood oxygen saturation (SatO2), obtained through pulse-oximetry, is an easy, non-invasive, and universally used clinical parameter that can provide real-time information about tissue oxygenation. However, since the oxygen–haemoglobin dissociation curve presents a S-shape, the SatO2 has a low sensitivity for the detection of hypoxaemia and might not closely correlate with the PaO2 [12]. Despite this, the SatO2/FiO2 ratio, which is calculated dividing the SatO2 by the FiO2, was shown to be closely correlated with the PaO2/FiO2 ratio, suggesting that the SatO2/FiO2 ratio could be used for the identification and follow-up of patients with ARDS avoiding arterial puncture and blood gas analysis [13]. The respiratory rate-OXygenation (ROX) index is another interesting and non-invasive index that has been recently proposed and validated to assess the effectiveness of HFNC treatment in patients with acute hypoxemic respiratory failure [14, 15]. The ROX index is calculated as the ratio of the SatO2/FiO2 ratio and respiratory rate (RR) and it might be a useful predictor of important outcomes, such as mortality or intubation.

Thus, the aim of the present study was to evaluate the clinical performance of non-invasive respiratory function indices, such as the ROX index and the SatO2/FiO2 ratio, as compared to that of the PaO2/FiO2 ratio, for the prediction of clinically relevant outcomes (death or intubation, whichever comes first) in hospitalized patients for COVID-19 pneumonia.

## Methods

### Study design

This is a four-centre retrospective cohort study conducted in patients hospitalized for COVID-19 pneumonia in medical and sub-intensive wards. We classified as medical wards both the Internal medicine and Liver Unit at Verona and Padua University Hospitals, whereas both the San Luca Hospital, Istituto Auxologico Italiano, in Milan and the Respiratory Intensive Care Unit in Mantua Hospital were considered as sub-intensive wards, since they were properly equipped to perform intensive monitoring and non-invasive ventilation. All the patients admitted to these wards between March and May 2020 were included. The study was approved as patients’ registry by the Ethical committee of all recruiting centres. All patients provided oral informed consent to be included in the study.

### Patients

Four hundred and fifty-six consecutive patients were finally included in the study. Inclusion criteria were: (1) hospital admission with a diagnosis of COVID-19 pneumonia based on the detection of SARS-CoV-2 RNA in nasopharyngeal swabs plus one between (a) either a chest X-ray showing pneumonia or (b) a chest computed tomography showing pneumonia, (c) or respiratory failure that required oxygen treatment [16]. Patients younger than 18 years, patients with an indication for immediate intubation, and those with a do-not-intubate order were excluded. Patients electively intubated for diagnostic or therapeutic procedures (e.g., bronchoscopy and surgery) were also excluded. A composite outcome of transfer to ICU or death (whichever comes first) within 28 days after hospital admission, was assessed. Patients transferred to ICU were those that required intubation and invasive mechanical ventilation.

### Medical history, laboratory data, device description and management

For each participant, anthropometric and demographic data were extracted from medical records as well as data about medical history and current medications. In detail, a complete medical history (including hypertension, diabetes, ischemic heart disease, heart failure, chronic obstructive pulmonary disease, chronic kidney disease and chronic liver disease) and medication use in the previous 14 days (including angiotensin receptor enzyme inhibitors, angiotensin receptor blockers, beta-blockers, antiplatelet agents and oral anticoagulants) were recorded. Data about laboratory tests performed during hospital admission were extracted and recorded. These included: haemoglobin, white blood cells, lymphocytes, platelets, C-reactive protein, d-dimer, serum creatinine, alanine aminotransferase, lactate dehydrogenase, creatine kinase and serum ferritin. Data about the SatO2, measured by pulse oximetry; the PaO2, derived from ABG test; the FiO2, predetermined in patients treated with Venturi masks, extrapolated in patients treated with nasal cannulas or reservoir bag masks, set in patients under NIV; were recorded and used to calculate the PaO2/FiO2 ratio, the SatO2/FiO2 ratio, and the ROX index within the first 24 h of hospital admission; in particular, the first data obtained on arrival at medical or sub-intensive wards was registered. The ROX index was calculated as the ratio of the SatO2/FiO2 ratio and RR as previously described [14, 15]. The decision for a single patient’s intubation was agreed upon after discussion between medical doctors in the medical or sub-intensive wards and from the ICU (Intensivists). It was based upon clinical criteria, including oxygen desaturation, increased RR, and arterial blood gas evaluation (including the PaO2/FiO2 ratio) indicating actual or incipient respiratory failure.

### Statistical analysis

Continuous variables are presented as mean ± standard deviation or median with interquartile range based on data distribution. Categorical variables are expressed as percentages. Either one-way ANOVA or Kruskal Wallis one-way ANOVA were used to compare continuous variables according to the data distribution pattern (normal or not normal). Categorical variables were compared using the Chi-square test. Logistic regression analyses were performed to determine if any anamnestic or clinical variables (age; sex; a history of either hypertension or diabetes; ALT; blood lymphocyte count; platelet count; levels of d-dimer, LDH or serum ferritin) could be independently associated with the study endpoint, a composite of admission to the ICU for intubation or death whichever came first. The variable selection was done through sequential replacement (a stepwise method) which consists of a combination of backward and forward techniques. If the *p* value was less than 0.05 or above 0.1 the covariates were, respectively, included and excluded from the regression model. No fixed variables were considered. The Area Under the Receiver Operator Characteristic Curves (AUROC) were performed to assess the capacity of PaO2/FiO2 ratio, SatO2/FiO2 ratio and ROX index for predicting the study endpoint. Furthermore, we tried to identify the optimal cutoff point of the PaO2/FiO2 ratio, SatO2/FiO2 ratio and ROX index for the prediction of the composite outcome. The main analysis was done according to the different intensity of care of the four centers: the medical wards (Verona and Padua) were compared to the sub-intensive wards (Milan and Mantua). Data about each centers considered separately are presented in the supplementary material. Statistical package for social science (SPSS) version 22 was used for all data analysis. All tests were two-sided, and *p* values < 0.05 were considered statistically significant.

## Results

**Fig. 1:**
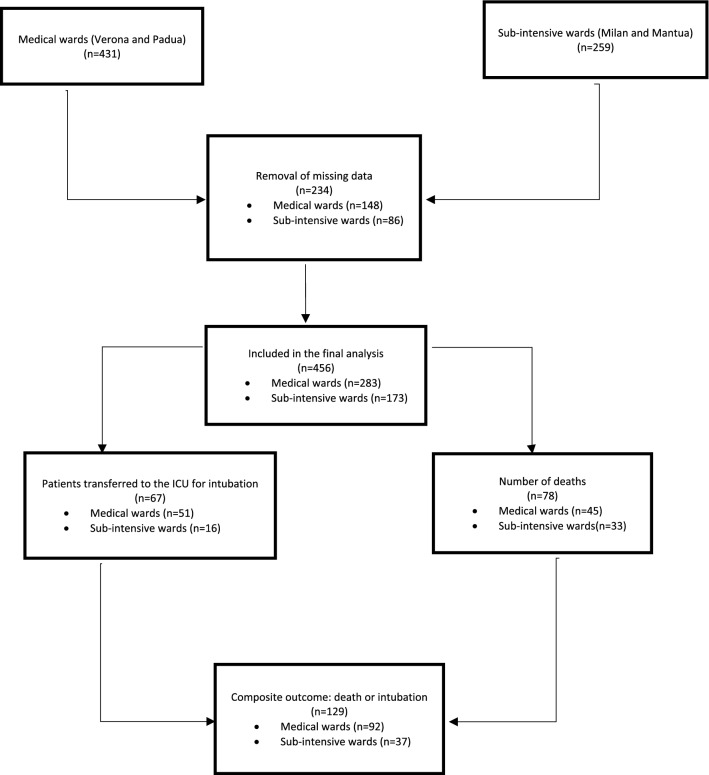
Patient’s flow diagram

Four hundred and fifty-six consecutive patients were enrolled. In the medical wards (Verona and Padua) 30 patients required NIV after hospital admission, while 51 patients were transferred to the ICU for intubation and 45 died. In the sub-intensive wards (Milan and Mantua) 121 patients underwent NIV during hospitalization, while the numbers of patients transferred to the ICU for intubation or deceased were 16 and 33, respectively (Fig. 1). Clinical and laboratory characteristics, according to the different intensity of care of the hospitalization wards, are presented in Table 1. In brief, patients hospitalized in the medical wards were older, while those admitted to the sub-intensive wards suffered from more severe forms of acute respiratory failure. Regarding biochemical variables, patients hospitalized in the sub-intensive wards presented higher levels of serum ferritin, lactate dehydrogenase, alanine aminotransferase, C-reactive protein and d-dimer. Furthermore, in the sub-intensive wards, a greater number of patients underwent NIV after admission. On the other hand, in this setting, the incidence of the composite outcome of transfer to the ICU for intubation or death was significantly lower than in the medical wards.

**Table 1:** Clinical and laboratory characteristics of patients at admission in the overall population, medical wards (Verona and Padua) and sub-intensive wards (Milan and Mantua)

	Overall population *N* = 456	Medical wards *N* = 283	Sub-intensive wards *N* = 173	*p* value
Age, years—M (IQR)	66 (55–77)	67 (56–79)	64 (54–73)	0.01
Gender, *n* (% of males)	310 (67.9)	178 (62.9)	132 (76.3)	n.s
PaO2/FiO2 ratio—M (IQR)	196.6 (129.3–313.8)	232.1 (160.6–323.8)	160.0 (116.0–243.6)	** < 0.0001**
ROX index—M (IQR)	12.9 (7.9–19.5)	15.0 (9.7–21.3)	9.2 (7.4–14.1)	** < 0.0001**
SatO2/FiO2 ratio—M (IQR)	276.5 (184.0–442.9)	333.3 (245.0–452.4)	195.4 (164.3–303.4)	** < 0.0001**
Drug use in the previous 14 days—*n* (%)				
ACE inhibitors	99 (21.7)	66 (23.3)	33 (19.1)	n.s
ARBs	81 (17.8)	48 (17.0)	33 (19.1)	n.s
Beta-blockers	91 (20.0)	61 (21.6)	30 (17.3)	n.s
Antiplatelet agents	68 (14.9)	38 (13.4)	30 (17.3)	n.s
Oral anticoagulants	36 (7.9)	30 (10.6)	6 (3.4)	**0.04**
Comorbidity—*n* (%)				
Hypertension	245 (53.7)	154 (54.4)	91 (52.6)	n.s
Diabetes	80 (17.5)	47 (16.6)	33 (19.1)	n.s
Heart failure	43 (9.4)	30 (10.6)	13 (7.5)	n.s
Chronic kidney disease	25 (5.5)	19 (6.7)	6 (3.5)	n.s
Chronic liver disease	16 (3.5)	16 (5.7)	0 (0)	**0.001**
Hb (g/dL)—m (SD)	13.1 ± 1.9	13.2 ± 1.9	13.0 ± 1.9	n.s
WBC (× 10^9^/L)—M (IQR)	6.1 (4.4–8.5)	5.7 (4.0–8.2)	6.7 (5.0–9.1)	**0.001**
Lymphocytes (× 10^9^/L)—M (IQR)	0.9 (0.7–1.3)	1.0 (0.7–1.2)	0.9 (0.6–1.3)	n.s
Platelets (× 10^9^/L)—M (IQR)	190 (155–225)	179 (144–231)	220 (163–297)	**0.001**
CRP (mg/L)—M (IQR)	91 (40–150)	70 (32–125)	127 (88–211)	** < 0.0001**
d-Dimer (µg/L)—M (IQR)	674 (182–1719)	247 (150–825)	1463 (867–3257)	** < 0.0001**
Serum creatinine (mg/dL)—M (IQR)	0.9 (0.7–1.2)	0.9 (0.7–1.2)	1.0 (0.8–1.1)	n.s
ALT (U/L)—M (IQR)	33 (21–52)	30 (20–48)	37 (22–59)	**0.01**
LDH (U/L)—M (IQR)	341 (264–507)	304 (251–402)	491 (318–735)	** < 0.0001**
CPK (U/L)—M (IQR)	109 (56–227)	114 (61–212)	96 (48–254)	n.s
Serum ferritin (μg/L)—M (IQR)	757 (384–1538)	727 (343–1245)	1012 (502–1803)	**0.004**
NIV after admission—*n* (%)	151 (33.1)	30 (10.6)	121 (69.9)	** < 0.0001**
Transfer to the ICU for intubation—(%)	67 (14.7)	51 (18.0)	16 (9.2)	** < 0.0001**
Mortality—(*n*%)	78 (17.1)	45 (15.9)	33 (19.1)	n.s
Transfer to the ICU for intubation or death—*n* (%)	129 (28.3)	92 (32.5)	37 (21.4)	**0.007**

Bold values indicate statistical significance

*m* mean; *SD* standard deviation; *M* median; *IQR* interquartile range; *n* number; *n.s* not significant; *PaO2* partial pressure of arterial oxygen; *FiO2* fraction of inspired oxygen; *SatO2* pulse oxygen saturation; *ACE* angiotensin converting enzyme; *ARB* angiotensin receptor blockers; *Hb* haemoglobin; *WBC* white blood cells; *CRP* C-reactive protein; *ALT* alanine aminotransferase; *LDH* lactate dehydrogenase; *CPK* creatine kinase; *NIV* non-invasive ventilation; *ICU* intensive care unit

Multiple logistic regression analysis showed that, both in the overall population and medical wards (Tables S1 and S2), the PaO2/FiO2 ratio, the ROX index and the SatO2/FiO2 ratio were all significantly associated with the composite outcome, whereas in the sub-intensive wards none of the three respiratory function indices was able to predict death or intubation (data not shown). In this setting, the only clinical variable that remained significantly associated with the composite outcome was age (data not shown).

**Fig. 2:**
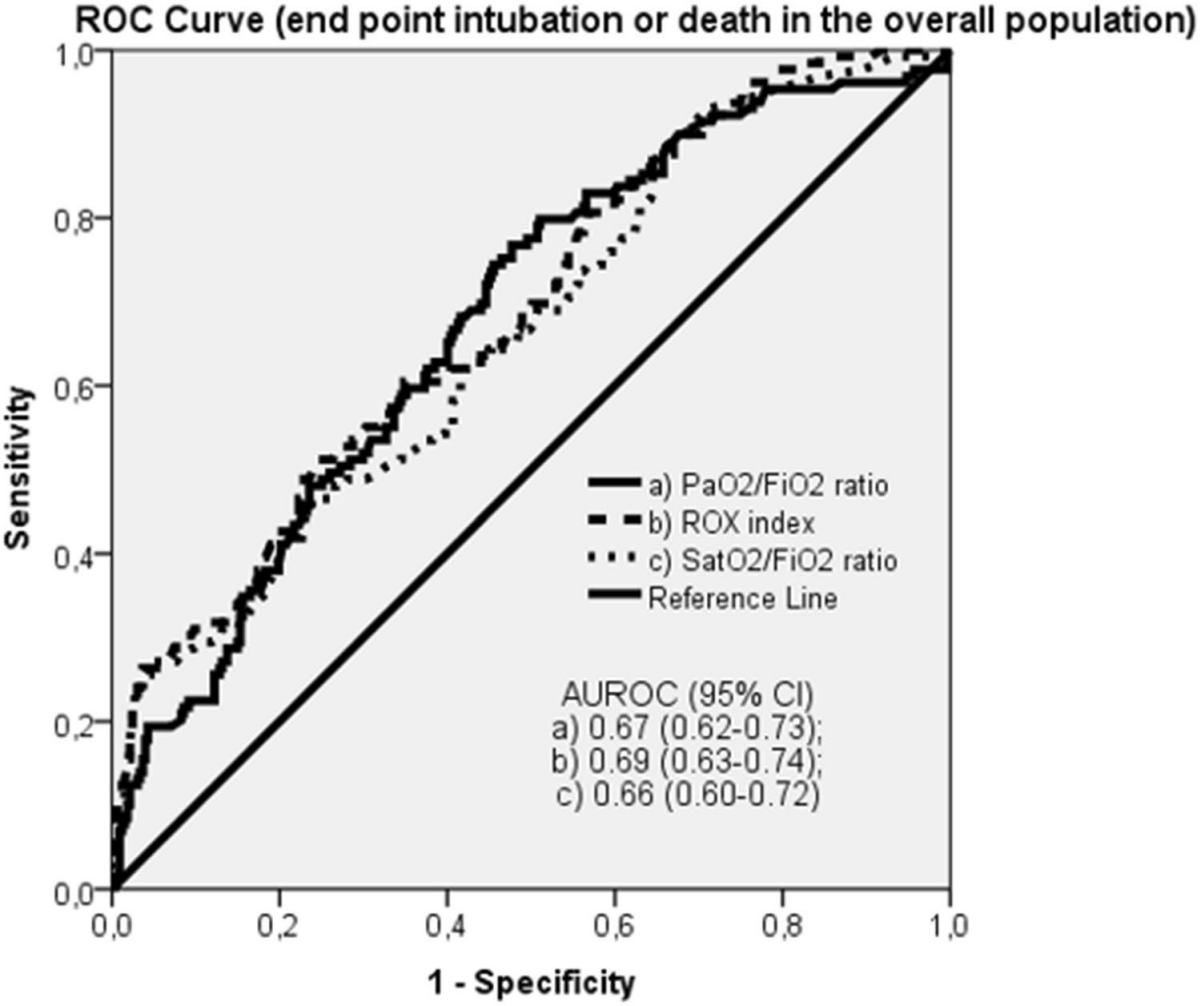
ROC curve: end point intubation or death in the overall population

**Fig. 3:**
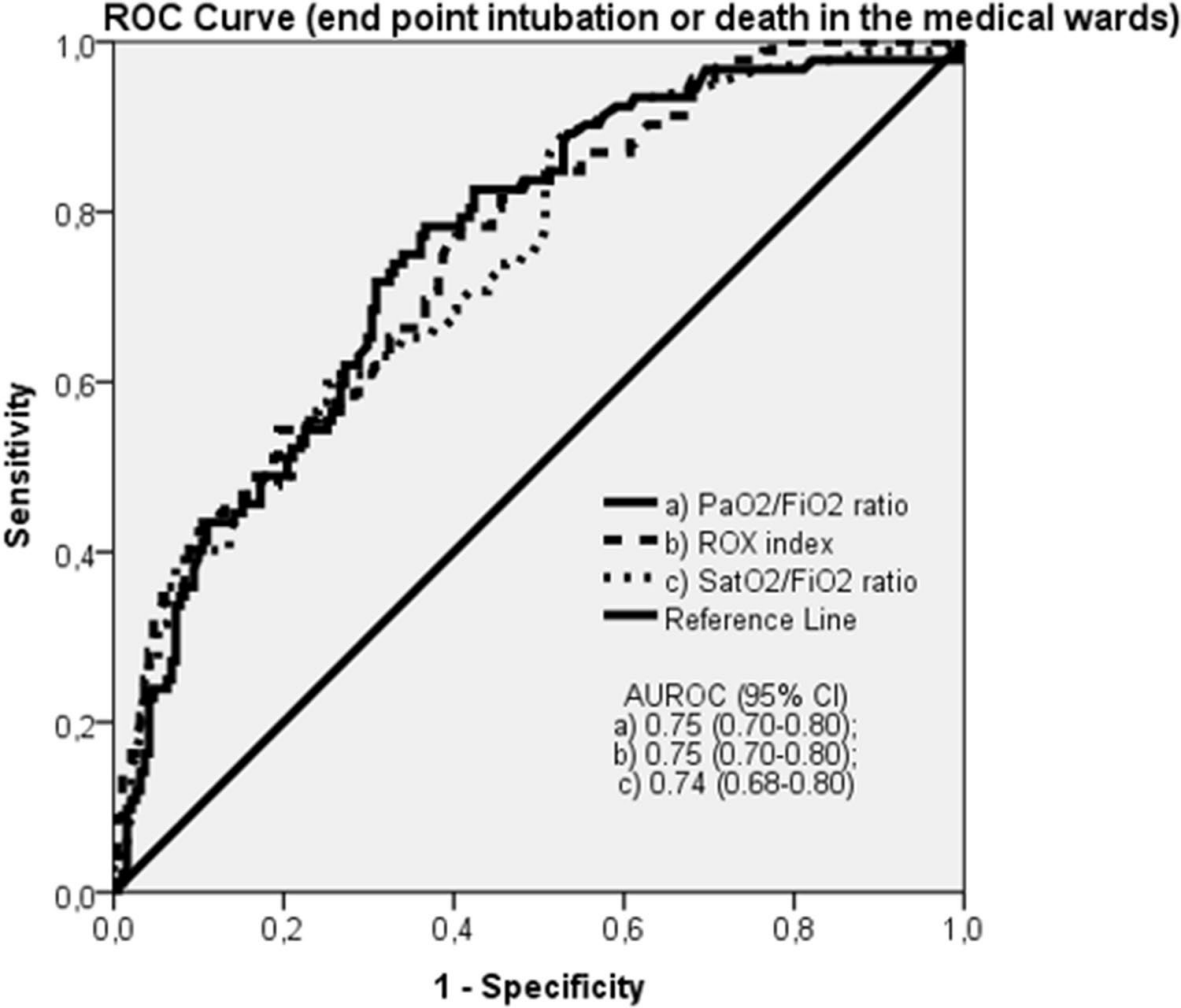
ROC curve: end point intubation or death in the medical wards

**Fig. 4:**
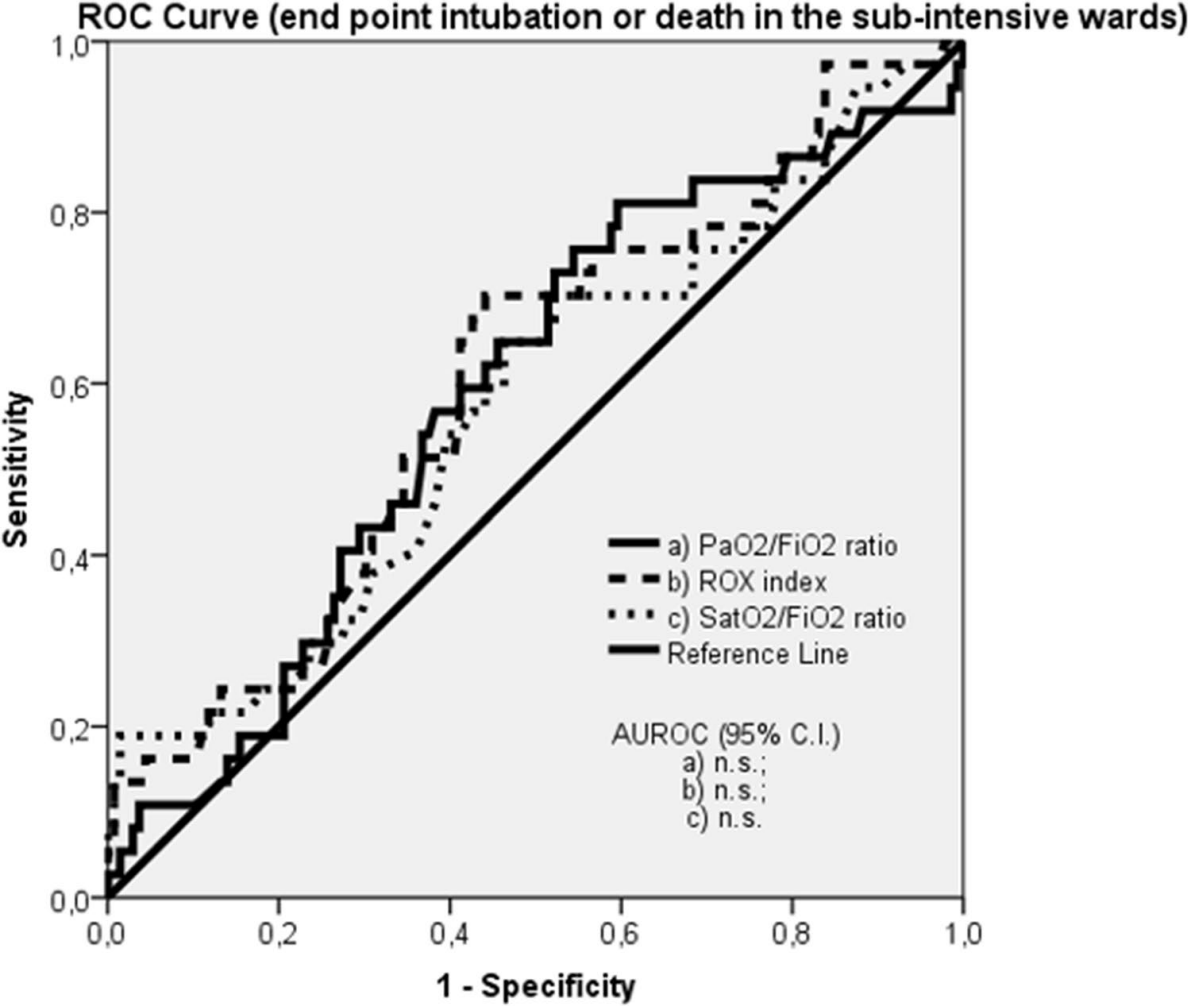
ROC curve: end point intubation or death in the sub-intensive wards

In the overall population the PaO2/FiO2 ratio, the ROX index and the SatO2/FiO2 ratio predicted admission to the ICU for intubation or death with an accuracy of 67%, 69% and 66%, respectively (Table 2 and Fig. 2), not significantly different between each others. The diagnostic performance of the three respiratory function indices resulted higher in the medical wards, where the transfer to the ICU for intubation or death were predicted by the PaO2/FiO2 ratio, the ROX index and the SatO2/FiO2 ratio with an accuracy of 75%, 75% and 74%, respectively, while was not significant in the sub-intensive wards (Table 2 and Figs. 3, 4).

**Table 2:** Discrimination ability of the PaO2/FiO2 ratio, ROX index and SatO2/FiO2 ratio in predicting transfer to the ICU for intubation or death in the overall population, medical wards (Verona and Padua) and sub-intensive wards (Milan and Mantua)

		AUROC	*p* value
Overall population *N* = 456	PaO2/FiO2 ratio	0.67 (0.62–0.73)	** < 0.0001**
	ROX index	0.69 (0.63–0.74)	** < 0.0001**
	SatO2/FiO2 ratio	0.66 (0.60–0.72)	** < 0.0001**
Medical wards *N* = 283	PaO2/FiO2 ratio	0.75 (0.70–0.80)	** < 0.0001**
	ROX index	0.75 (0.70–0.80)	** < 0.0001**
	SatO2/FiO2 ratio	0.74 (0.68–0.80)	** < 0.0001**
Sub-intensive wards *N* = 173	PaO2/FiO2 ratio	0.59 (0.49–0.69)	n.s
	ROX index	0.60 (0.50–0.70)	n.s
	SatO2/FiO2 ratio	0.57 (0.47–0.68)	n.s

Bold values indicate statistical significance

*ICU* intensive care unit; *AUROC* Area Under the Receiver Operating Characteristics; *n.s* not significant; *PaO2* partial pressure of arterial oxygen; *FiO2* fraction of inspired oxygen; *SatO2* pulse oxygen saturation

Traditionally the PaO2/FiO2 ratio cutoffs of 300 mmHg, 200 mmHg and 100 mmHg are used to identify mild, moderate, or severe ARDS, respectively [11]. In the medical wards, these cutoffs for the composite outcome resulted in a sensitivity of 85%, 65%, and 22% and a specificity of 35%, 70%, and 95%, respectively. The same levels of sensitivity and specificity, as for the three cutoffs of the PaO2/FiO2 ratio, could be obtained in the medical wards with ROX index values of 17.9, 13.7 and 4.0; and SatO2/FiO2 ratio values of 422.6, 295.4 and 114.6 (Table 3). The sensibility and specificity for the composite outcome in the overall population of the three PaO2/FiO2 ratio cutoffs and the corresponding values of ROX index and SatO2/FiO2 ratio are also presented in Table 3.

**Table 3:** Comparison between diagnostic performance of PaO2/FiO2 ratio, ROX index and SatO2/FiO2 ratio in predicting transfer to the ICU for intubation or death and relative cutoffs in the overall population, medical wards (Verona and Padua) and sub-intensive wards (Milan and Mantua)

			ROX index	SatO2/FiO2 ratio
Overall population *N* = 456	PaO2/FiO2 ratio = 300	Se 85%	17.7	415.5
		Sp 35%		
	PaO2/FiO2 ratio = 200	Se 70%	13.5	286.8
		Sp 56%		
	PaO2/FiO2 ratio = 100	Se 20%	5.3	122.6
		Sp 92%		
Medical wards *N* = 283	PaO2/FiO2 ratio = 300	Se 85%	17.9	422.6
		Sp 50%		
	PaO2/FiO2 ratio = 200	Se 65%	13.7	295.4
		Sp 70%		
	PaO2/FiO2 ratio = 100	Se 22%	4	114.6
		Sp 95%		
Sub-intensive wards *N* = 173	PaO2/FiO2 ratio = 300	n.s	–	–
	PaO2/FiO2 ratio = 200	n.s	–	–
	PaO2/FiO2 ratio = 100	n.s	–	–

*ICU* intensive care unit; *n.s* not significant; *PaO2* partial pressure of arterial oxygen; *FiO2* fraction of inspired oxygen; *SatO2* pulse oxygen saturation; *Se* sensibility; *Sp* specificity

Apart from the respiratory indices, the other covariates that remained independently associated with the composite outcome in all logistic models, both in the overall population and medical wards, were age and blood lymphocyte count. In the overall analysis, also being hospitalized in Mantua and serum levels of lactate dehydrogenase were statistically associated with the composite outcome (Table S1), whereas in the medical wards another significant covariate for the outcome of intubation or death was serum levels of alanine aminotransferase (Table S2). The adjustment for these covariates, when significant, highly increased the ability of the PaO2/FiO2 ratio, the ROX index and the SatO2/FiO2 ratio to predict transfer to the ICU for intubation or death (Table 4).

**Table 4:** Discrimination ability of the PaO2/FiO2 ratio, ROX index and SatO2/FiO2 ratio in predicting transfer to the ICU for intubation or death in the overall population and medical wards (Verona and Padua) after adjustment for age, blood lymphocyte count, alanine aminotransferases and care departments, when significant

		AUROC	*p* value
Overall population *N* = 456	PaO2/FiO2 ratio	0.84 (0.79–0.88)	** < 0.0001**
	ROX index	0.84 (0.79–0.89)	** < 0.0001**
	SatO2/FiO2 ratio	0.83 (0.78–0.88)	** < 0.0001**
Medical wards *N* = 283	PaO2/FiO2 ratio	0.81 (0.75–0.88)	** < 0.0001**
	ROX index	0.82 (0.76–0.88)	** < 0.0001**
	SatO2/FiO2 ratio	0.81 (0.75–0.87)	** < 0.0001**

Bold values indicate statistical significance

*ICU* intensive care unit; *AUROC* Area Under the Receiver Operating Characteristics; *n.s* not significant; *PaO2* partial pressure of arterial oxygen; *FiO2* fraction of inspired oxygen; *SatO2* pulse oxygen saturation

The secondary analysis, comparing the different Units, showed results in line with the main one (Supplementary material, Tables S3–S5).

## Discussion

The results of our multicentric study of 456 hospitalized patients for COVID-19 pneumonia showed an acceptable performance of two non-invasive and easily measurable respiratory indices in predicting transfer to ICU or death. Interestingly, in medical wards, the prediction accuracy of the ROX index and the SatO2/FiO2 ratio was comparable to that of the PaO2/FiO2 ratio, which is considered the gold standard for the diagnosis of respiratory failure and ARDS in clinical practice.

Unfortunately, frequent and repeated ABG tests are not always feasible in medical wards. This occurred, especially, during COVID-19 outbreak when hospitals withstood unrelenting pressure due to the continuous inflow of patients with SARS-CoV-2 infection and acute respiratory failure that required constant monitoring of the respiratory function. In this dramatic scenario, the availability of respiratory indices which allow a rapid and non-invasive evaluation of disease severity and treatments efficacy may help clinicians to promptly identify those patients at higher risk of intubation and death.

There is a growing interest in the validation of non-invasive respiratory indices, such as the ROX index, as easy-to-apply prognostic tools in COVID-19 pneumonia. In the last few months, a couple of studies were set to investigate whether the ROX index could predict the effectiveness of HFNC treatment for SARS-CoV-2 infection [17, 18]. Furthermore, Zaboli and colleagues applied the ROX index at triage assessment of 273 patients who presented to the emergency department for dyspnoea due to SARS-CoV-2 infection. The study aimed to explore the prognostic ability of the ROX index in predicting ARDS development or need for intubation within 72 h after triage evaluation. They found median ROX index values at the admission of 13.1 and 15.3 in those that either subsequently required intubation or were diagnosed with ARDS, respectively. The ROX index showed good accuracy in predicting ARDS development with an AUROC of 0.845 [19]. These results are in line with those by Suliman and colleagues, where the ROX index at admission was one of the independent predictors of intubation [20]. In another Italian study, the ROX index measured at admission in the emergency department was a strong predictor not only of subsequent hospitalization but also of higher 30-day mortality. In particular, ROX index values < 22.3 were found to be significantly related to higher mortality with an AUROC of 0.764 [21].

In contrast with previous investigations, our multicentric study is the first that assessed the performance of the ROX index and the SatO2/FiO2 ratio compared to the gold standard PaO2/FiO2 ratio invasive index. We found that not only the traditional PaO2/FiO2 ratio but also the ROX index and the SatO2/FiO2 ratio were highly significantly associated with the composite outcome of transfer to ICU for intubation or death. Furthermore, the prognostic accuracy of the ROX index and the SatO2/FiO2 ratio was non-inferior to that of PaO2/FiO2 ratio and, especially in the medical wards, was associated with the outcome. Instead, in the sub-intensive wards of Milan and Mantua hospitals, the association between these indices and the outcome was not statistically significant. Thus, the performance of the ROX index and the other respiratory indices can partly depend on the kind of patients admitted in wards with different intensities of care. The differences regard both the disease severity in the admitted patients and the devices available to support respiratory function during the first wave of SARS-CoV-2 infection in Italy. In fact, the indices of respiratory function, the percentage of patients using NIV support as well as the inflammatory biomarkers, such as levels of serum ferritin, LDH, d-dimer and lymphocytes were significantly higher in the sub-intensive wards, as compared to the medical ones indicating a more severe form of respiratory failure and systemic inflammation in these patients.

Finally, our study identifies different values of the ROX index and the SatO2/FiO2 ratio that correspond to the PaO2/FiO2 ratio cutoffs conventionally used to identify higher severity of respiratory failure. These non-invasive indices might allow for early identification of those patients who could benefit from more aggressive interventions, such as an earlier transfer to the ICU and intubation.

The present study has several limitations. Its retrospective and observational design does not allow to draw any definitive conclusion about the actual interchangeability of different indices of respiratory distress. Moreover, some biochemical tests were lacking or were available in restricted sub-groups of patients, which did not allow us to consider them in our analyses. Even if to date this appears to be the largest database evaluating the ROX index as a prognostic tool in COVID-19 pneumonia, the sample size of our study is somehow limited, when data from each medical Unit are analysed separately. The inclusion of both low-intensity and medium-intensity care Units and the multicentre design of our study could be considered a strength, since they allowed for meaningful comparisons of different settings of care, but also a weakness, since other differences among Centres in the care of patients could have in some way influenced the results. Many other studies evaluated different clinical, laboratory, and imaging markers in their ability to predict outcomes. Indeed, many risk scores were proposed but most of them did not include the respiratory indices that we have considered [22–24]. Our study does not propose another prognostic model to predict the outcome of patients affected by COVID-19 pneumonia, but rather shows that both non-invasive and invasive indices of respiratory distress have similar accuracy in predicting the clinical outcomes of COVID-19 patients admitted to medical wards.

In conclusion, the ROX index and the SatO2/FiO2 ratio demonstrated, in medical wards, an acceptable accuracy in the identification of hospitalized patients for SARS-CoV-2 infection at higher risk of death or transfer to the ICU for intubation. The non-invasiveness of the ROX index and the SatO2/FiO2 ratio, their simplicity and good reproducibility, could make them excellent prognostic and monitoring tools, especially in medical wards, where repeated ABG tests are not always feasible.

## Supplementary Information

Below is the link to the electronic supplementary material.

Supplementary file1 (DOCX 37 KB)

## References

[1] European Centre for Disease Prevention and Control. Novel coronavirus disease 2019 (COVID-19) pandemic: increased transmission in the EU/EEA and the UK—sixth update—12 March 2020. Stockholm: ECDC; 2020. Novel coronavirus disease 2019 (COVID-19) pandemic : increased transmission in the EU/EEA and the UK—sixth update. Rapid Risk Assess. 2020;2019(March).

[2] Chen N, Zhou M, Dong X, Qu J, Gong F, Han Y (2020). Epidemiological and clinical characteristics of 99 cases of 2019 novel coronavirus pneumonia in Wuhan, China: a descriptive study. Lancet.

[3] Gattinoni L, Chiumello D, Rossi S (2020). COVID-19 pneumonia: ARDS or not?. Crit Care.

[4] Guan W, Ni Z, Hu Y, Liang W, Ou C, He J (2020). Clinical characteristics of coronavirus disease 2019 in China. N Engl J Med.

[5] Onder G, Rezza G, Brusaferro S (2020). Case-fatality rate and characteristics of patients dying in relation to COVID-19 in Italy. J Am Med Assoc.

[6] Immovilli P, Morelli N, Antonucci E, Radaelli G, Barbera M, Guidetti D (2020). COVID-19 mortality and ICU admission: the Italian experience. Crit Care.

[7] Grasselli G, Zangrillo A, Zanella A, Antonelli M, Cabrini L, Castelli A (2020). Baseline characteristics and outcomes of 1591 patients infected with SARS-CoV-2 admitted to ICUs of the lombardy region, Italy. J Am Med Assoc.

[8] Istituto Superiore di Sanità (ISS). Characteristics of SARS-CoV-2 patients dying in Italy Report based on available data on May 21st, 2020 1. 2020;4–8. Available from: https://www.epicentro.iss.it/en/coronavirus/bollettino/Report-COVID-2019_21_may_2020.pdf

[9] Villar J, Blanco J, Del Campo R, Andaluz-Ojeda D, Díaz-Domínguez FJ, Muriel A (2015). Assessment of PaO2/FiO2 for stratification of patients with moderate and severe acute respiratory distress syndrome. BMJ Open.

[10] Whiting J, Edriss H, Yang S, Nugent K (2016). Peak pressures and PaO2/FiO2 ratios are associated with adverse outcomes in patients on mechanical ventilators. Am J Med Sci.

[11] Ranieri VM, Rubenfeld GD, Thompson BT, Ferguson ND, Caldwell E, Fan E (2012). Acute respiratory distress syndrome: the Berlin definition. J Am Med Assoc.

[12] Jubran A (2004). Pulse oximetry. Intensive Care Med.

[13] Rice TW, Wheeler AP, Bernard GR, Hayden DL, Schoenfeld DA, Ware LB (2007). Comparison of the SpO2/FIO2 ratio and the PaO 2/FIO2 ratio in patients with acute lung injury or ARDS. Chest.

[14] Roca O, Messika J, Caralt B, García-de-Acilu M, Sztrymf B, Ricard JD (2016). Predicting success of high-flow nasal cannula in pneumonia patients with hypoxemic respiratory failure: the utility of the ROX index. J Crit Care.

[15] Roca O, Caralt B, Messika J, Samper M, Sztrymf B, Hernández G (2019). An index combining respiratory rate and oxygenation to predict outcome of nasal high-flow therapy. Am J Respir Crit Care Med.

[16] Sethuraman N, Jeremiah SS, Ryo A (2020). Interpreting diagnostic tests for SARS-CoV-2. J Am Med Assoc.

[17] Hu M, Zhou Q, Zheng R, Li X, Ling J, Chen Y (2020). Application of high-flow nasal cannula in hypoxemic patients with COVID-19: a retrospective cohort study. BMC Pulm Med.

[18] Chandel A, Patolia S, Brown AW, Collins AC, Sahjwani D, Khangoora V (2020). High-flow nasal cannula therapy in COVID-19: using the ROX index to predict success. Respir Care.

[19] Zaboli A, Ausserhofer D, Pfeifer N, Sibilio S, Tezza G, Ciccariello L, et al. The ROX index can be a useful tool for the triage evaluation of COVID-19 patients with dyspnoea. J Adv Nurs [Internet]. 2021;(November 2020):1–9. Available from: http://www.ncbi.nlm.nih.gov/pubmed/33792953 10.1111/jan.14848 PMC8251286 33792953

[20] Suliman LA, Abdelgawad TT, Farrag NS, Abdelwahab HW (2021). Validity of rox index in prediction of risk of intubation in patients with covid-19 pneumonia. Adv Respir Med.

[21] Gianstefani A, Farina G, Salvatore V, Alvau F, Artesiani ML, Bonfatti S (2021). Role of ROX index in the first assessment of COVID-19 patients in the emergency department. Intern Emerg Med.

[22] Wynants L, Van Calster B, Collins GS, Riley RD, Heinze G, Schuit E et al (2021) Prediction models for diagnosis and prognosis of covid-19: Systematic review and critical appraisal. BMJ 16:1959–1965 10.1136/bmj.m1328 PMC7222643 32265220

[23] Liu Y, Gao W, Guo W, Guo Y, Shi M, Dong G (2020). Prominent coagulation disorder is closely related to inflammatory response and could be as a prognostic indicator for ICU patients with COVID-19. J Thromb Thrombolysis.

[24] Toussie D, Voutsinas N, Finkelstein M, Cedillo MA, Manna S, Maron SZ (2020). Clinical and chest radiography features determine patient outcomes in young and middle-aged adults with COVID-19. Radiology.

