# Supplementary material for: Performance of non-invasive respiratory function indices in predicting clinical outcomes in patients hospitalized for COVID-19 pneumonia in medical and sub-intensive wards: a retrospective cohort study
Source: Intern Emerg Med. 2022 Jan 28;17(4):1097–106. doi: 10.1007/s11739-021-02922-6 (PMC8796181; doi:10.1007/s11739-021-02922-6)
Supplement: Supplementary file 1 — Supplementary file1 (DOCX 37 KB) [file 11739_2021_2922_MOESM1_ESM.docx]

|  | **beta** | **SEM** | **Exp(B), CI 95%** | **p-value** | **R^2^** |  |
| --- | --- | --- | --- | --- | --- | --- |
| **Age (years)** | **ICU or death** | 0.05 | 0.01 | 1.05 (1.02-1.08) | **0.0001** | 0.39 |
| **Hospitalized in Mantua** |  | -2.71 | 0.61 | 0.66 (0.02-0.21) | **0.0001** |  |
| **PaO2/FiO2 ratio** |  | -0.05 | 0.02 | 0.995 (0.992-0.998) | **0.005** |  |
| **Lymphocytes (x10^9^/L)** |  | -1.46 | 0.40 | 0.23 (0.10-0.51) | **0.01** |  |
| **LDH (U/L)** |  | 0.002 | 0.001 | 1.002 (1.001-1.003) | **0.02** |  |

**Tables S1. Determinants of transfer to the ICU for intubation or death in stepwise multivariate logistic regression analysis in the overall population.**

|  | **beta** | **SEM** | **Exp(B), CI 95%** | **p-value** | **R^2^** |  |
| --- | --- | --- | --- | --- | --- | --- |
| **Age (years)** | **ICU or death** | 0.05 | 0.01 | 1.05 (1.02-1.08) | **<0.0001** | 0.40 |
| **Hospitalized in Mantua** |  | -2.62 | 0.59 | 0.07 (0.02-0.23) | **<0.0001** |  |
| **ROX index** |  | -0.08 | 0.02 | 0.92 (0.88-0.96) | **0.001** |  |
| **Lymphocytes (x10^9^/L)** |  | -1.43 | 0.40 | 0.24 (0.10-0.53) | **0.0001** |  |
| **LDH (U/L)** |  | 0.02 | 0.01 | 1.02 (1.01-1.03) | **0.003** |  |

|  | **beta** | **SEM** | **Exp(B), CI 95%** | **p-value** | **R^2^** |  |
| --- | --- | --- | --- | --- | --- | --- |
| **Age (years)** | **ICU or death** | 0.05 | 0.01 | 1.04 (1.02-1.08) | **<0.0001** | 0.38 |
| **Hospitalized in Mantua** |  | -2.69 | 0.61 | 0.07 (0.02-0.23) | **<0.0001** |  |
| **SatO2/FiO2 ratio** |  | -0.03 | 0.01 | 0.996 (0.994-0.999) | **0.03** |  |
| **Lymphocytes (x10^9^/L)** |  | -1.46 | 0.40 | 0.23 (0.10-0.51) | **0.0001** |  |
| **LDH (U/L)** |  | 0.02 | 0.006 | 1.02 (1.01-1.03) | **0.02** |  |

Legend: PaO2, partial pressure of arterial oxygen; FiO2, fraction of inspired oxygen; SatO2, pulse oxygen saturation; LDH, lactate dehydrogenase; ICU, intensive care unit.

**Tables S2. Determinants of transfer to the ICU for intubation or death in stepwise multivariate logistic regression analysis in the medical wards (Verona and Padua).**

|  | **beta** | **SEM** | **Exp(B), CI 95%** | **p-value** | **R^2^** |  |
| --- | --- | --- | --- | --- | --- | --- |
| **Age (years)** | **ICU or death** | 0.05 | 0.01 | 1.05 (1.02-1.08) | **0.001** | 0.35 |
| **PaO2/FiO2 ratio** |  | -0.04 | 0.02 | 0.996 (0.992-0.998) | **0.009** |  |
| **Lymphocytes (x10^9^/L)** |  | -1.16 | 0.46 | 0.31 (0.13-0.76) | **0.01** |  |
| **ALT (U/L)** |  | 0.02 | 0.007 | 1.02 (1.01-1.03) | **0.003** |  |

|  | **beta** | **SEM** | **Exp(B), CI 95%** | **p-value** | **R^2^** |  |
| --- | --- | --- | --- | --- | --- | --- |
| **Age (years)** | **ICU or death** | 0.05 | 0.01 | 1.05 (1.02-1.08) | **<0.0001** | 0.40 |
| **ROX index** |  | -0.08 | 0.03 | 0.93 (0.88-0.96) | **0.009** |  |
| **Lymphocytes (x10^9^/L)** |  | -1.09 | 0.45 | 0.33 (0.14-0.82) | **0.01** |  |
| **ALT (U/L)** |  | 0.02 | 0.01 | 1.02 (1.01-1.03) | **0.002** |  |

|  | **beta** | **SEM** | **Exp(B), CI 95%** | **p-value** | **R^2^** |  |
| --- | --- | --- | --- | --- | --- | --- |
| **Age** | **ICU or death** | 0.04 | 0.01 | 1.04 (1.02-1.07) | **0.001** | 0.39 |
| **SatO2/FiO2 ratio** |  | -0.04 | 0.01 | 0.996 (0.994-0.999) | **0.01** |  |
| **Lymphocytes (x10^9^/L)** |  | -1.16 | 0.46 | 0.31 (0.14-0.70) | **0.005** |  |
| **ALT (U/L)** |  | 0.02 | 0.006 | 1.02 (1.01-1.03) | **0.02** |  |

Legend: PaO2, partial pressure of arterial oxygen; FiO2, fraction of inspired oxygen; SatO2, pulse oxygen saturation; ALT, alanine aminotransferase; ICU, intensive care unit.

**Table S3. Patients clinical and laboratory characteristics at admission according to the hospital.**

|  | **Verona N=73** | **Padua N=210** | **Milan N=79** | **Mantua N=94** | **p-value** |
| --- | --- | --- | --- | --- | --- |
| **Age, ys – M (IQR), m (SD)** | 73 (55-81) | 64±15 | 67±14 | 60±13 | **<0.001*** |
| **Gender, n (% of males)** | 45 (61.6) | 133 (63.3) | 60 (75.9) | 72 (76.6) | n.s. |
| **SatO2/FiO2 ratio – M (IQR)** | 340.7 (258.7-457.1) | 329.3 (231.9-452.3) | 274.2 (184.0-442.8) | 182.0 (158.3-235.0) | **<0.001*** |
| **PaO2/FiO2 ratio – M (IQR)** | 221.4 (140.0-304.8) | 242.9 (164.8-332.9) | 220.0 (145.1-323.8) | 126.9 (106.9-180.1) | **<0.001*** |
| **ROX index – M (IQR)** | 15.6 (9.8-21.3) | 14.8 (9.5-21.4) | 13.9 (7.9-21.8) | 8.1 (7.1-10.8) | **<0.001*** |
| **Drug use in the previous 14 days – n (%)**  *ACE inhibitors*  *ARBs*  *Beta-blockers*  *Antiplatelet agents*  *Oral anticoagulants* | 23 (31.5)  7 (9.6)  17 (23.3)  11 (15.1)  9 (12.3) | 43 (20.5)  41 (19.5)  44 (21.0)  27 (12.9)  21 (10.0) | 16 (20.3)  15 (19)  13 (16.5)  16 (20.3)  4 (5) | 17 (18.1)  18 (19.1)  17 (18.1)  14 (14.9)  2 (2.1) | n.s.  n.s.  n.s.  n.s.  n.s. |
| **Comorbidity – n (%)**  *Hypertension*  *Diabetes*  *Heart failure*  *Chronic pulmonary obstructive disease*  *Chronic kidney disease*  *Chronic liver disease* | 43 (58.9)  10 (13.7)  7 (9.6)  4 (5.5)  8 (11.0)  1 (1.4) | 111 (52.9)  37 (17.6)  23 (11.0)  18 (8.6)  11 (5.2)  15 (7.1) | 45 (57)  13 (16.5)  9 (11.4)  8 (10.1)  3 (3.8)  0 (0) | 46 (48.8)  20 (21.3)  4 (4.3)  n.a.  3 (3.2)  0 (0) | n.s.  n.s.  n.s.  -  n.s.  **0.001§** |
| **Haemoglobin (g/dL) – M (IQR)** | 13.2 (11.2-14.5) | 13.4 (12.4-14.5) | 12.8 (11.7-14.5) | 13.1 (12.0-14.1) | n.s. |
| **WBC (x10^9^/L) – M (IQR)** | 6.95 (4.3-9.2) | 5.4 (3.9-7.9) | 6.4 (4.9-8.7) | 6.6 (5.9-9.3) | n.s. |
| **Lymphocytes (x10^9^/L) – M (IQR)** | 0.83 (0.7-1.1) | 1.0 (0.7-1.3) | 1.2 (0.7-1.5) | 0.8 (0.6-1.1) | n.s. |
| **Platelets (x10^9^/L) – M (IQR)** | 182 (140-233) | 175 (144-230) | 231 (159-333) | 216 (167-276) | **0.002#** |
| **C-reactive protein (mg/L) – M (IQR)** | 73.5 (31-116.8) | 68.5 (33.7-130.0) | n.a. | 127.6 (88.0-211.9) | - |
| **D-Dimer (µg/L) – M (IQR)** | 1666 (1026-2548) | 1870 (1500-3520) | 1653 (973-3340) | 1286 (799-3195) | n.s. |
| **Serum creatinine (mg/dL) – M (IQR)** | 0.96 (0.78-1.2) | 0.90 (0.76-1.08) | n.a. | 0.97 (0.79-1.16) | - |
| **ALT (U/L) – M (IQR)** | 28 (18-43) | 31 (21-49) | 41 (24-59) | 35 (21-59) | n.s. |
| **LDH (U/L) – M (IQR)** | 303 (260-359) | 307 (240-404) | 306 (249-398) | 666 (527-902) | **<0.001*** |
| **CPK (U/L) – M (IQR)** | 108 (54-250) | 119 (66-211) | n.a. | 96 (48-254) | - |
| **Serum ferritin (μg/L) – M (IQR)** | 554 (252-1320) | 742 (378-1232) | 762 (547-1546) | 1086 (430-2294) | **0.03**** |
| **NIV after admission – n (%)** | 0 (0) | 30 (14.3) | 27 (34.2) | 94 (100) | **<0.001^*, §, #,^^** |
| **ICU or death – n (%)** | 27 (37.0) | 65 (31.0) | 24 (30.4) | 13 (13.8) | **<0.001*** |

Legend: m, mean; SD, standard deviation; M, median; IQR, interquartile range; n, number; ACE, angiotensin converting enzyme; ARB, angiotensin receptor blockers; PaO2, partial pressure of arterial oxygen; FiO2, fraction of inspired oxygen; SatO2, pulse oxygen saturation; WBC, white blood cells; ALT, alanine aminotransferase; LDH, lactate dehydrogenase; CPK, creatine kinase; NIV, non-invasive ventilation; ICU, intensive care unit.

* Mantua significantly different compared to Verona, Padua and Milan; **Mantua significantly different compared to Verona and Padua; #Milan significantly different compared to Verona, Padua and Mantua; §Padua significantly different compared to Verona, Milan and Mantua. ^Verona significantly different compared to Padua, Milan and Mantua

**Table S4. Discrimination ability of the PaO2/FiO2 ratio, ROX index and SatO2/FiO2 ratio in predicting transfer to the ICU for intubation or death according to the hospital.**

|  | | **AUROC** | **p-value** |
| --- | --- | --- | --- |
| **Verona**  **N=73** | **PaO2/FiO2 ratio** | 0.84 (0.75-0.93) | **<0.001** |
|  | **ROX index** | 0.80 (0.70-0.90) | **<0.001** |
|  | **SatO2/FiO2 ratio** | 0.79 (0.68-0.90) | **<0.001** |
| **Padua**  **N=210** | **PaO2/FiO2 ratio** | 0.79 (0.72-0.85) | **<0.001** |
|  | **ROX index** | 0.74 (0.67-0.81) | **<0.001** |
|  | **SatO2/FiO2 ratio** | 0.74 (0.66-0.81) | **<0.001** |
| **Milan**  **N=79** | **PaO2/FiO2 ratio** | 0.71 (0.57-0.85) | **0.004** |
|  | **ROX index** | 0.72 (0.60-0.84) | **0.002** |
|  | **SatO2/FiO2 ratio** | 0.69 (0.56-0.82) | **0.008** |
| **Mantua**  **N=94** | **PaO2/FiO2 ratio** | 0.53 (0.41-0.65) | n.s. |
|  | **ROX index** | 0.58 (0.45-0.71) | n.s. |
|  | **SatO2/FiO2 ratio** | 0.49 (0.36-0.62) | n.s. |

Legend: ICU, intensive care unit; AUROC, Area Under the Receiver Operating Characteristics; n.s, not significant; PaO2, partial pressure of arterial oxygen; FiO2, fraction of inspired oxygen; SatO2, pulse oxygen saturation.

**Table S5. Comparison between diagnostic performance of the PaO2/FiO2 ratio, ROX index and SatO2/FiO2 ratio in predicting transfer to the ICU for intubation or death and relative cut-offs according to the hospital.**

|  | | | **ROX index** | **SatO2/FiO2 ratio** |
| --- | --- | --- | --- | --- |
| **Verona**  **N=73** | **PaO2/FiO2 ratio = 300** | Se 96% | 20 | 422 |
|  |  | Sp 42% | 22.9 | 459 |
|  | **PaO2/FiO2 ratio = 200** | Se 70% | 14.7 | 333 |
|  |  | Sp 70% | 15.4 | 320 |
|  | **PaO2/FiO2 ratio = 100** | Se 33% | 8.5 | 234 |
|  |  | Sp 98% | 6.6 | 210 |
| **Padua N=210** | **PaO2/FiO2 ratio = 300** | Se 81% | 16.8 | 430 |
|  |  | Sp 61% | 14.8 | 324 |
|  | **PaO2/FiO2 ratio = 200** | Se 55% | 12.7 | 266 |
|  |  | Sp 88% | 9.4 | 212 |
|  | **PaO2/FiO2 ratio = 100** | Se 17% | 3.7 | 112 |
|  |  | Sp 97% | 3.7 | 112 |
| **Milan**  **N=79** | **PaO2/FiO2 ratio = 300** | Se 79% | 17.9 | 426 |
|  |  | Sp 36% | 21.2 | 440 |
|  | **PaO2/FiO2 ratio = 200** | Se 70% | 14.6 | 320 |
|  |  | Sp 70% | 12.1 | 237 |
|  | **PaO2/FiO2 ratio = 100** | Se 20% | 5.8 | 124 |
|  |  | Sp 98% | 5.9 | 117 |
| **Mantua N=94** | **PaO2/FiO2 ratio = 300** | - | - | - |
|  | **PaO2/FiO2 ratio = 200** | - | - | - |
|  | **PaO2/FiO2 ratio = 100** | - | - | - |

Legend: ICU, intensive care unit; n.s, not significant; PaO2, partial pressure of arterial oxygen; FiO2, fraction of inspired oxygen; SatO2, pulse oxygen saturation; Se, sensibility; Sp, specificity
